# Good science, poor visibility? Communicating behavioural nutrition and physical activity evidence in the algorithmic attention economy

**DOI:** 10.1186/s12966-026-01962-5

**Published:** 2026-10-06

**Authors:** Stephen Barrett, Emma Ostermeier-Karim, Jennifer Gale, Kylie Wilson, Michael Wheeler, Courtney Anne West, Emily Denniss, António Palmeira

**Affiliations:** 1 Allied Health Directorate, Bendigo Health, Bendigo, VIC 3550 Australia; 2 Holsworth Biomedical Research Centre, La Trobe University, Bendigo, VIC 3552 Australia; 3 College of Rehabilitation Sciences, University of Manitoba, Winnipeg, MB Canada; 4 School of Sport, Exercise and Health, Human Potential Research Institute, Auckland University of Technology, Auckland, New Zealand; 5 University of Arizona Cancer Center, Tucson, AZ USA; 6 Institute for Physical Activity and Nutrition, Deakin University, Melbourne, VIC Australia; 7 School of Biosciences, Faculty of Science, University of Melbourne, Melbourne, VIC Australia; 8 Faculty of Medicine and Health, University of Sydney, Sydney, NSW Australia; 9 Institute for Musculoskeletal Health, Sydney Local Health District, Sydney, NSW Australia; 10 Institute for Physical Activity and Nutrition, School of Health and Social Development, Deakin University, Geelong, Australia; 11 CIDEFES Universidade Lusofona, Lisbon, Portugal; 12 CIFI2D Universidade do Porto, Porto, Portugal

**Keywords:** Behavioural nutrition, Digital dissemination, Algorithmic visibility, Science communication, Knowledge translation

## Abstract

Behavioural nutrition and physical activity research has produced a strong and growing evidence base to inform prevention, treatment, and policy across a wide range of health outcomes. Yet the visibility of this evidence beyond academic audiences does not consistently reflect its scientific quality. Increasingly, dissemination occurs within digital environments shaped by algorithms that prioritise engagement, simplicity, novelty, and emotional salience over nuance and uncertainty. This creates a mismatch between the complexity that characterises behaviour change science and the attention-driven logics that shape what evidence becomes visible.

This Comment was prompted by our involvement in developing the NESI (Network of Early Career Researchers and Students of ISBNPA) Podcast and ASPA (Asia-Pacific Society for Physical Activity) Radio, initiatives designed to disseminate credible behavioural science. Despite prioritising high-quality, peer-reviewed research, we encountered persistent challenges in achieving visibility relative to simplified or commercially framed health narratives amplified in digital spaces.

We argue that visibility in digital environments is algorithmically mediated in ways that can systematically disadvantage complex, context-dependent evidence characteristic of behavioural nutrition and physical activity research. We explore the risks of equating visibility with impact, including oversimplification and erosion of trust, while contending that disengagement from contemporary communication platforms is not viable for a field committed to translation. We propose reframing dissemination as a collective responsibility of researchers, journals, and professional societies, focused on stewardship, integrity, and principled engagement. Addressing the visibility gap is essential to ensuring that credible evidence remains visible, interpretable, and influential within an attention-driven information ecosystem.

## The digital “visibility gap” in behavioural nutrition and physical activity evidence

Behavioural nutrition and physical activity research occupies a paradoxical position: the evidence base has never been stronger, yet sustained visibility beyond academia remains limited. The field has produced robust evidence demonstrating the effectiveness of interventions across individual, community, and policy levels; however, this evidence often remains insufficiently visible to non-academic audiences, including the general public and policymakers. While the visibility gap has long been recognised, it is becoming more pronounced as dissemination increasingly occurs within digitally mediated environments shaped by algorithmic curation [1, 2].

These digital environments include social media, search engines, online news aggregators, and AI-mediated recommendation systems. Content visibility is shaped less by chronology or authority than by algorithms designed to maximise engagement and time on platforms [3]. Visibility is therefore influenced more by clicks and engagement than by information credibility [3]. For behavioural nutrition and physical activity research, evidence now reaches professional and public audiences through systems whose incentives are not aligned with scientific norms.

Against this backdrop, we use “visibility” to refer not simply to audience size, but to the probability that credible evidence is encountered by relevant professional and public audiences. In contemporary digital environments, such exposure is not proportional to information quality [3].

Behavioural science is inherently complex [4]. Interventions are multi-component, effects are context-dependent, and outcomes are probabilistic rather than deterministic [4]. These features are essential to scientific integrity, and preserving this nuance is central to responsible communication. However, this complexity aligns poorly with digital environments that reward brevity, certainty, novelty, and emotional resonance. Consistent with this, recent evidence demonstrates that misinformation relating to physical activity and nutrition is prevalent across social media platforms [5, 6]. As a result, evidence-based messages are often overshadowed by simplified, commercial, or misleading narratives about diet, exercise, and health. Evidence that fails to reach practitioners, policymakers, and communities cannot meaningfully influence behaviour or systems, regardless of its methodological strength [7]. The assumption that evidence will “speak for itself” is increasingly untenable in digital environments, where visibility is actively shaped rather than passively accrued. When credible evidence remains less visible than more engaging but less rigorous alternatives, opportunities for policy influence and systems-level change are diminished.

## Platform dynamics and the algorithmic attention economy

Digital environments do not merely host information; they actively shape what is seen, shared, and amplified [1]. Algorithms prioritise engagement, often irrespective of accuracy or information quality [2, 3]. These dynamics can elevate misleading or oversimplified health information, challenging the circulation of credible behavioural research [1–3].

These dynamics are particularly consequential for behavioural nutrition and physical activity research, where achieving impact typically requires sustained behaviour change across multiple interacting components and contexts [4]. Unlike biomedical interventions that may be communicated through singular effect estimates or discrete outcomes, behavioural interventions often require explanations of mechanisms, theoretical underpinnings, heterogeneity of response, and implementation context, features that are poorly accommodated by digital environments [8].

We do not suggest that dissemination outcomes are fully determined by algorithms. Rather, we suggest that algorithmic curation introduces systematic tendencies shaping which forms of health information gain prominence. Over time, such tendencies can distort public and professional understandings of credible behavioural evidence.

Importantly, because digital environments personalise exposure in response to users’ interactions and engagement patterns, these distortions are unlikely to be evenly distributed. Different information environments may expose some groups to greater volumes of low-quality health content, potentially exacerbating inequities in access to credible behavioural nutrition and physical activity evidence.

## Insights from multimedia dissemination initiatives

Professional societies and early-career networks have increasingly experimented with multimedia dissemination formats, including podcasts, webinars, and social media engagement to broaden access to behavioural science beyond traditional academic channels [7]. The NESI (Network of Early Career Researchers and Students of ISBNPA) Podcast and ASPA (Asia-Pacific Society for Physical Activity) Radio were developed to amplify credible behavioural science, prioritising methodological transparency, and global representation. Our experiences with these initiatives highlighted a persistent tension: content grounded in evidence and uncertainty frequently struggled to achieve visibility relative to more simplified or commercially framed health narratives circulating within the same digital environments. These patterns were not explained by content quality alone, but by platform dynamics shaping discoverability.

Multimedia formats such as podcasts can support depth and dialogue that are difficult to achieve through traditional dissemination channels. Nevertheless, producing and sustaining high-quality content requires substantial time and often unpaid labour. Visibility remains contingent on algorithms, networks, and promotional capacity; effort and quality alone do not guarantee exposure.

These experiences highlight our central point: challenges in disseminating evidence cannot be reduced to individual communication skills or researcher motivation. They reflect structural features of digital environments that shape which evidence is seen and shared. Early-career researchers, researchers from low- and middle-income settings where health burdens are disproportionately high, and those working outside dominant institutional networks may experience compounded visibility barriers, raising important equity considerations for the field.

## The risks of equating visibility with impact

As awareness of the visibility gap grows, so too does the temptation to equate visibility with impact. However, the uncritical pursuit of visibility carries risks. Simplifying messages to maximise visibility can erode nuance, overstate effects, or understate uncertainty. For example, visual abstracts and infographics are increasingly being used to summarise research, increase social media engagement and boost Altmetric scores, yet some have been found to present insufficient context or misleading interpretations and may be viewed as a substitute for reading the full-text [9]. Over time, such distortions may undermine trust in authoritative experts and behavioural science. In an era of misinformation and potentially declining trust in institutions, behavioural science cannot trade integrity for attention [8].

Crucially, this is not an argument against engagement, but against false dichotomies. The challenge is not choosing between rigour and visibility, but developing deliberate, values-driven approaches to communicating evidence in digital spaces shaped by attention-based incentives. Despite growing interest in science communication, relatively little evidence exists regarding which dissemination strategies are most effective [7]. Future research should evaluate which communication strategies, message characteristics, and platforms most effectively increase meaningful engagement with behavioural science and promote behaviour change, while also examining the extent to which increased visibility translates into policy, practice, and health outcomes.

## From passive publishing to active stewardship

Journals, universities and professional societies occupy a pivotal position in addressing these challenges. Traditionally focused on gatekeeping within academic ecosystems, they now face the need for active stewardship after publication. Science benefits from established systems of quality control, including peer review and editorial oversight that regulate how evidence is generated and evaluated. In contrast, the communication of science beyond formal publication channels remains comparatively unstructured and individually driven [3]. As health information and research increasingly circulate through podcasts, social media, and other digital formats, the norms and safeguards for responsible dissemination are less clearly defined [3]. This governance gap matters because visibility in contemporary environments can shape public understanding and trust as much as the evidence itself. Compounding this challenge, end-users often have limited means of assessing the credibility of health information encountered within algorithmically curated environments, increasing the importance of trusted institutions acting as stewards of evidence [3, 10]. This is not a call to compete with algorithmic optimisation, but to ensure that communication remains anchored in scientific norms even as evidence enters mediated public spaces.

This stewardship could build on existing efforts by some journals and professional societies, including editorially supported multimedia summaries, society-led amplification of key findings, and capacity-building and training initiatives that equip researchers to communicate evidence responsibly and persuasively within contemporary digital environments. Expanding these efforts more systematically may strengthen the visibility of credible evidence in algorithmically mediated environments. Rather than prescribing platforms, we advocate a principles-based approach to dissemination stewardship, prioritising accuracy, proportionality, and collective amplification over individual visibility. Framing dissemination as a shared institutional responsibility may help safeguard the integrity of behavioural nutrition and physical activity evidence as it circulates within algorithmically mediated environments.

## Reframing impact

Impact is not only a function of citations or policy documents, but of whether evidence is encountered, understood, and trusted by those who can act on it. The longstanding challenges of making research visible beyond academia have been intensified by a rapidly evolving media landscape, including the expansion of digital platforms, misinformation, and AI systems. Although these environments often amplify simplified content, they also create opportunities for meaningful dissemination of high-quality evidence. Behavioural researchers must engage strategically and responsibly within these digital environments to ensure that rigorous evidence gains visibility among broader audiences, while communicating in ways that are accessible and align with audience values without sacrificing scientific nuance.

The contribution of this Comment is to reframe visibility as a structural determinant of impact. We argue that dissemination through digital environments should be intentional, ethical, and institutionally supported to ensure that behavioural science is visible among the public, policymakers and practitioners.

Science is not finished until it is communicated. Addressing the visibility gap is not about chasing virality, but about safeguarding credible evidence in an information ecosystem increasingly shaped by attention rather than accuracy. Maintaining the integrity, interpretability, visibility and trustworthiness of behavioural evidence should therefore be considered a core component of scientific responsibility.

## Conclusions

Behavioural nutrition and physical activity science faces a growing mismatch between how evidence is generated and how it circulates in digital environments. Algorithms shape visibility in ways that can marginalise nuanced, evidence-based messages, with implications for translation and impact. Rather than retreating from these spaces or uncritically adapting to them, the field must develop principled approaches to dissemination that balance visibility with rigour. Journals, professional societies, and researchers each have a role to play in stewarding evidence in the public sphere. In an attention-driven economy, maintaining the visibility of good science is not optional; it is a matter of scientific responsibility.

## Data Availability

No datasets were generated or analysed during the current study.
